# Subcellular Localization and Activity of TRPM4 in Medial Prefrontal Cortex Layer 2/3

**DOI:** 10.3389/fncel.2018.00012

**Published:** 2018-01-30

**Authors:** Denise Riquelme, Ian Silva, Ashleigh M. Philp, Juan P. Huidobro-Toro, Oscar Cerda, James S. Trimmer, Elias Leiva-Salcedo

**Affiliations:** ^1^Departamento de Biología, Facultad de Química y Biología, Universidad de Santiago de Chile, Santiago, Chile; ^2^Programa de Biología Celular y Molecular, Instituto de Ciencias Biomédicas, Facultad de Medicina, Universidad de Chile, Santiago, Chile; ^3^Department of Neurobiology, Physiology and Behavior, College of Biological Sciences, University of California, Davis, Davis, CA, United States; ^4^Centro para el Desarrollo de Nanociencias y Nanotecnología, Santiago, Chile; ^5^Millennium Nucleus of Ion Channels-Associated Diseases (MiNICAD), Santiago, Chile; ^6^Department of Physiology and Membrane Biology, School of Medicine, University of California, Davis, Davis CA, United States

**Keywords:** TRPM4, perforated patch, medial prefrontal cortex (mPFC), layer 2/3 pyramidal neuron, postnatal development

## Abstract

TRPM4 is a Ca^2+^-activated non-selective cationic channel that conducts monovalent cations. TRPM4 has been proposed to contribute to burst firing and sustained activity in several brain regions, however, the cellular and subcellular pattern of TRPM4 expression in medial prefrontal cortex (mPFC) during postnatal development has not been elucidated. Here, we use multiplex immunofluorescence labeling of brain sections to characterize the postnatal developmental expression of TRPM4 in the mouse mPFC. We also performed electrophysiological recordings to correlate the expression of TRPM4 immunoreactivity with the presence of TRPM4-like currents. We found that TRPM4 is expressed from the first postnatal day, with expression increasing up to postnatal day 35. Additionally, in perforated patch clamp experiments, we found that TRPM4-like currents were active at resting membrane potentials at all postnatal ages studied. Moreover, TRPM4 is expressed in both pyramidal neurons and interneurons. TRPM4 expression is localized in the soma and proximal dendrites, but not in the axon initial segment of pyramidal neurons. This subcellular localization is consistent with a reduction in the basal current only when we locally perfused 9-Phenanthrol in the soma, but not upon perfusion in the medial or distal dendrites. Our results show a specific localization of TRPM4 expression in neurons in the mPFC and that a 9-Phenanthrol sensitive current is active at resting membrane potential, suggesting specific functional roles in mPFC neurons during postnatal development and in adulthood.

## Introduction

The prefrontal cortex (PFC) is a highly-evolved brain area that participates in higher cognitive functions such as short-term memory, decision-making and executive function (Miller, 2000). As other brain areas, the PFC displays extensive development and maturation, and during postnatal development a series of structural and functional changes occur that increase neuronal connectivity, synaptic strength, and excitability (Casey et al., 2000; Tsujimoto, 2008). Several types of neurons in layers 2/3 and 5 of the PFC show persistent firing after a transient electrical stimulus, cholinergic activity or mGluR activation, and it has been suggested that this persistent activity underlies various aspects of PFC function (Takehara-Nishiuchi and McNaughton, 2008; Arnsten et al., 2012; Siegel et al., 2012; Fenton et al., 2014). Two mechanisms appear to be involved in persistent firing: reverberating circuits through recurrent synaptic connectivity (Wang, 1999; Teramae and Fukai, 2005; Okamoto and Fukai, 2009; Knauer et al., 2013) and the nature of intrinsic excitability as mediated by the expression and activity of different ion channels (McCormick et al., 2003; Tahvildari et al., 2008; Ben-Mabrouk and Tryba, 2010; Boehlen et al., 2013; Papoutsi et al., 2013).

Ion conductances such as persistent sodium current (*I_NaP_*) (Tazerart et al., 2008), hyperpolarization-activated current (*I_h_*) (Thuault et al., 2013), and calcium-activated non-selective cation (CAN) current (*I_CAN_*) (Yoshida et al., 2008) play major roles in neuronal excitability, intrinsic firing patterns and persistent activity. *T*ransient *R*eceptor *P*otential type *M*elastatin 4 (TRPM4) is a non-selective cation channel activated by [Ca^2+^]_i_, and that is permeable to monovalent cations, impermeable to divalent cations, and is thought to underlay the CAN current in many cells (Launay et al., 2002; Vennekens and Nilius, 2007; Guinamard et al., 2010).

Interestingly, several brain areas express TRPM4, where it has been proposed to participate in neuronal functions related to burst firing, persistent activity, and rhythmic firing. For instance, TRPM4 participates in the transient plateau potential and neuronal bursting induced by mGluR type I activation in the preBötzinger nucleus (Mironov, 2013). Similarly, TRPM4 is involved in the slow afterdepolarizing current induced by strong depolarization, a key mechanism for sustained neuronal firing in Purkinje neurons in lobe IX of the cerebellum (Kim et al., 2013). In the hypothalamus, TRPM4 expressed in supraoptic and periventricular nuclei seems to contribute to the depolarizing afterpotential and phasic bursting (Teruyama et al., 2011). TRPM4 expression in *substantia nigra pars compacta* may play an important role in the plateau potential and burst firing of dopaminergic neurons (Mrejeru et al., 2011). Additionally, TRPM4 participates in the dendritic depolarization necessary to induce certain forms of LTP in the hippocampal CA1 area (Menigoz et al., 2016). Recently, Lei et al. (2014) demonstrated that TRPM4 is expressed in pyramidal neurons of layer 5 in mouse mPFC. However, no detailed description in other layers, nor its specific subcellular localization pattern, has been reported. A better understanding of the cellular expression and subcellular localization of TRPM4 is crucial for defining its function in cortical networks. In this work, we showed that in mice, immunolabeling for TRPM4 is present in mPFC from the first postnatal day, and its presence correlated with a 9-Phenanthrol sensitive CAN current compatible with TRPM4. TRPM4 is expressed in both pyramidal neurons and interneurons. Additionally, pyramidal neurons in the mPFC exhibit TRPM4 immunolabeling and function that is confined to the soma and proximal dendrites, while interneuron expression is mainly somatic. These data support a role for TRPM4 in the physiology of diverse populations of neurons in mouse mPFC layer 2/3.

## Materials and Methods

### Animals and Tissue Sectioning

All experiments were conducted following the animal protocols approved by the Ethical Committee of the Universidad de Santiago de Chile following the rules and guidelines from the Chilean Council of Science and Technology (CONICYT). Briefly, male C57BL/6J mice were housed in temperature and humidity controlled facility with a 12/12 h light/dark cycle with water and food *ad libitum*.

Coronal brain slices containing mPFC were prepared from mice at postnatal days (P) 0, 7, 14, 35, and 90. Briefly, mice were deeply anesthetized by isoflurane inhalation; mice at P35 and P90 were intracardially perfused with ice-cold PBS 0.1 M (50 mL). The mice were decapitated and brains were quickly removed and placed in 4% w/v formaldehyde (freshly prepared from paraformaldehyde, Merck, 818715), in PBS 0.1 M pH 7.4, and incubated overnight at 4°C. The brains were then placed in a vibrating tissue slicer (1000 Plus, Vibratome), and sectioning was performed using a sapphire blade to obtain 60 μm thick slices.

### Immunoblot Analyses

Immunoblot experiments were conducted by following the protocol described in Cáceres et al. (2015). Briefly, transfected HEK293 cells were washed once with ice-cold DPBS and lysed with 150 μL of ice-cold lysis buffer containing 1% (v/v) Triton X-100 (Merck, 108603), 150 mM NaCl, 1 mM EDTA, 50 mM Tris-HCl (pH 7.4), 1 mM sodium orthovanadate, 5 mM NaF, 1 mM phenylmethylsulfonyl fluoride (PMSF; Sigma, 78830), and protease inhibitor cocktail (Cytoskeleton, Inc., PIC02) for 10 min at 4°C. The lysates were centrifuged at 12,000 *g* at 4°C for 10 min. The supernatants were mixed with 150 μL of 2x Reducing Sample Buffer [RSB: 60 mM Tris-HCl pH 6.8, 25% (v/v) glycerol, 2% (w/v) SDS (Sigma, L5750), 14.4 mM 2-mercaptoethanol, 0.1% (w/v) bromophenol blue] and size-fractionated by 7.5% SDS-PAGE. Lauryl sulfate (Sigma, L5750) was the form of SDS used in all gel solutions. The immunoblots were visualized by Pierce ECL Western Blotting Substrate (ThermoFisher, 34080) and images were acquired with a MiniHD9 imager (Uvitec).

### Anti-TRPM4 Monoclonal Antibody Generation and Characterization

The anti-TRPM4 mAb L88/86 was generated against the GST-tagged human TRPM4 (UniProt ID Q8TD43) carboxyl-terminal region (GST-cTRPM4) as described (Bekele-Arcuri et al., 1996). GST-cTRPM4 for monoclonal antibody generation was generated by PCR-amplification of the region corresponding to amino acids 1040–1214 using pcDNA4TO-FLAG-hTRPM4 (Launay et al., 2002) plasmid as a template. The PCR insert was cloned into the BamHI/XhoI sites of pGEX-KG plasmid. The GST-cTRPM4 fusion protein was purified from the *E. coli* BL21/DE3 strain. Purified GST-cTRPM4 protein was used to immunize two BALB/c mice. Serum immunoreactivity against GST-cTRPM4 was evaluated by enzyme-linked immunosorbant assay (ELISA) (Bekele-Arcuri et al., 1996). The mouse that presented higher TRPM4 immunoreactivity was euthanized for splenectomy. Dissociated splenocytes were fused to SP2/0 mouse myeloma cells as described (Trimmer et al., 1985). Hybridomas were selected for growth media in HAT media and were screened by ELISA using GST-cTRPM4-coated plates. Positive clones were tested by immunoblot and immunofluorescence assays (Supplementary Figures 1A–C).

### Primary Antibodies

Two different rabbit polyclonal antibodies against TRPM4, ACC-044 (RRID:AB_2040250) and ab104572 (RRID:AB_10712148), were obtained from Alomone (Israel) and Abcam (United States) respectively. Monoclonal anti-TRPM4 L88/86 hybridoma tissue culture supernatant (RRID: AB_2716758). Monoclonal anti-TRPM4 clone 10H5 (RRID: AB_2208624) was obtained from Origene (United States). Mouse monoclonal and rabbit polyclonal anti-MAP2 antibodies (ab11267, RRID:AB_297885; and ab32454, RRID:AB_776174, respectively) were obtained from Abcam (United States). Anti-NeuN (MAB377, RRID:AB_2298772), anti-GAD67 (MAB5406, RRID:AB_2278725) mouse monoclonal antibodies, and the anti-Neurogranin (AB5620, RRID:AB_2171427) rabbit polyclonal antibody were obtained from Millipore (United States). Anti-AnkG (75-146, RRID:AB_10673030) mouse monoclonal antibody N106/36 was obtained from the UC Davis/NIH NeuroMab Facility (United States), and rabbit polyclonal anti-FLAG (F7425, RRID:AB_439687) was obtained from Sigma (United States) (**Table 1**).

**Table 1:** List of antibodies.

Antibody	Isotype	Dilution	Final [ ], μg/mL	Source	Type	Catalog#	RRDI	Purification
Rabbit anti-TRPM4	IgG	1:50	17	Alomone	P	ACC-044	AB_2040250	AP
Rabbit anti-TRPM4	IgG	1:100	10	Abcam	P	ab104572	AB_10712148	IF
Mouse anti-TRPM4	IgM	Non-diluted supernatant			M		AB_2716758	
Mouse anti-TRPM4	IgG1	1:50	15	Origene	M	10H5	AB_2208624	Asc
Mouse anti-NeuN	IgG1	1:300	3	Millipore	M	MAB377	AB_2298772	AP
Mouse anti-GAD67	IgG2a	1:1000	1	Millipore	M	MAB5406	AB_2278725	AP
Rabbit anti-Neurogranin	IgG	1:500	2	Millipore	P	AB5620	AB_91937	AP
Mouse anti-Ankyrin G	IgG2a	1:100	8	Neuromab	M	75-146	AB_10673030	AP
Mouse anti-MAP2	IgG1	1:200	12	Abcam	M	ab11267	AB_297885	AP
Rabbit anti-MAP2	IgG	1:300	3	Abcam	P	ab32454	AB_776174	AP
Rabbit anti-FLAG	IgG	1:100	8	Sigma–Aldrich	P	F7425	AB_439687	AP
Alexa Fluor 546 conjugated goat anti-mouse IgG1	IgG	1:2000	1	ThermoFisher Scientific	P	A21123	AB_2535765	AP
Alexa Fluor 546 conjugated goat anti-mouse IgG2a	IgG	1:2000	1	ThermoFisher Scientific	P	A21133	AB_2535772	AP
Alexa Fluor 488 conjugated donkey anti-rabbit IgG	IgG	1:2000	1	ThermoFisher Scientific	P	A21206	AB_2535792	AP
Alexa Fluor 405 conjugated goat anti-rabbit IgG	IgG	1:2000	1	ThermoFisher Scientific	P	A31556	AB_221605	AP
Alexa Fluor 488 conjugated goat anti-mouse IgM	IgG	1:2000	1	ThermoFisher Scientific	P	A21042	AB_2535711	AP
Alexa Fluor 647-conjugated goat anti-mouse IgG	IgG	1:2000	1	ThermoFisher Scientific	P	A21235	AB_2535804	AP


*P: polyclonal; M: monoclonal; AP: affinity purified; Asc: purified from mouse ascites fluids by affinity chromatography; IF: IgG fraction.*

### Secondary Antibodies

Alexa Fluor 546 goat anti-mouse IgG1 (A21123), Alexa Fluor 546 goat anti-mouse IgG2a (A21133), Alexa Fluor 488 donkey anti-rabbit (A21206), Alexa Fluor 405 goat anti-rabbit (A31556), Alexa Fluor 488 goat anti-mouse IgM (A21042), and Alexa Fluor 647 goat anti-mouse IgG (A21235) were obtained from Life Technologies (**Table 1**).

### Immunofluorescence Labeling of Mouse Brain Sections

Mouse brain sections containing the mPFC were blocked in 10% v/v normal goat serum (NGS, Sigma, G9023) in 0.1 M PBS, pH 7.4 for 1 h at room temperature (RT); then sections were incubated with primary antibody diluted in the same NGS solution using gentle rocking overnight at 4°C. Sections were washed in 0.1 M PBS for 30 min before incubation with the secondary antibody (1.5 h at RT). After six washes of 5 min each with PBS, sections were mounted on glass slides using Mowiol mounting media and covered with a 0.17 mm thick coverslip. Fluorescence was observed in a LSM-510 or LSM-800 inverted confocal microscope (Zeiss) using 10x 0.3 N.A. and 40x 1.4 N.A. magnification objectives, a scan speed of 6 and a scan average of 4. Images used for full area composition were acquired using a 10x, 0.3 N.A. objective.

### Image Analysis

All images were obtained at 8 or 16-bit pixel depth at 2048 × 2048 resolution. For panoramic view, single images were shading corrected using Zen 14 (Zeiss) and assembled into image arrays covering the cortical region using the photomerge function in Adobe Photoshop CC (Adobe). Images were adjusted for overall brightness, contrast, and levels. Image analyzes were performed using Fiji-ImageJ. Data were collected and analyzed from at least four sections from four animals.

### Cell Culture and shRNA Transfection

B16-F10 and COS-7 cells were grown in DMEM (Sigma, D5648) supplemented with 10% v/v FBS (Gibco, 10437-028), 100 U/mL penicillin, and 100 μg/mL streptomycin (Gibco, 15140-122) in a controlled atmosphere incubator at 37°C in 5% CO_2_. Cells were transfected with FUGW plasmid containing a shRNA sequence against mouse TRPM4 [kindly provided by Dr. David J. Linden, Johns Hopkins University School of Medicine (Kim et al., 2013)] using Lipofectamine 2000 (Invitrogen, 11668-027) following the manufacturer’s instructions.

### Cell Immunofluorescence

Cells grown on 12 mm coverslips were fixed 10 min in 4% w/v formaldehyde (freshly prepared from paraformaldehyde) in 0.01 M PBS pH 7.4 and then washed three times in PBS. Cells were permeabilized 10 min with 0.1% v/v Triton X-100 in PBS, then blocked with NGS for 1 h at RT. Cells were incubated overnight at 4°C with primary antibodies diluted in the same blocking solution, after the incubation, cells were washed three times for 5 min each with PBS before incubation with the secondary antibody (1.5 h at RT). After three washes of 5 min each with PBS, cells were mounted with Prolong Gold mounting media (Thermofisher, P36934). Fluorescence was observed in a LSM-510 or LSM-800 inverted confocal microscope (Zeiss) using 40x 1.4 N.A. magnification objectives, a scan speed of 6 and a scan average of 4.

### Electrophysiological Recordings

Mice (C57BL/6J) between postnatal days 7 to 90 (see specific ages in results) were deeply anesthetized with isoflurane and their brains were quickly removed and placed in ice-cold oxygenated (95% O_2_, 5% CO_2_) high-magnesium artificial cerebrospinal fluid (ACSF) containing (in mM): 124 NaCl, 2.5 KCl, 5 MgCl_2_, 0.5 CaCl_2_, 1.25 NaH_2_PO_4_, 0.4 ascorbic acid, 2 sodium pyruvate, 25 NaHCO_3_, 11 glucose, pH 7.4. Tissue blocks containing PFC were placed in a vibratome to obtain parasagittal brain slices (350 μm thick). Then, slices were transferred to a chamber containing oxygenated ACSF containing (in mM): 125 NaCl, 2.5 KCl, 1.3 MgCl_2_, 2.5 CaCl_2_, 1.25 NaH_2_PO_4_, 25 NaHCO_3_, 11 Glucose, pH 7.4. After 1.5 h of recovery, slices were transferred to a recording chamber mounted on a Axioscope 200FS DIC microscope (Zeiss). Slices were continuously perfused with oxygenated ACSF (2–3 mL/min) at 32 ± 2°C.

#### Voltage-Clamp Experiments

Whole-cell recordings were performed from layer 2/3 pyramidal neurons in the mPFC using borosilicate glass pipettes (4 to 6 GΩ; Sutter, BF150) filled with intracellular solution containing (in mM): 120 CsCH_3_SO_3_, 10 CsCl, 10 HEPES, 5 TEA-Cl, 0.5 EGTA (unless otherwise indicated), 2 Mg-ATP, 0.3 Na-GTP, 5 QX-314, pH 7.2 adjusted with CsOH (∼300 mOsm). For perforated-patch protocols, 300 μg/mL nystatin (Sigma, N4014) diluted in DMSO (Sigma, D8418) was added to the intracellular solutions. A stable access resistance (R_a_) was typically obtained after 10 to 15 min (R_a_ = 10–20 MΩ) (Spruston and Johnston, 1992). In the case of a sudden decrease in the access resistance, the recording was discarded. Pipette and whole-cell capacitance as well as series resistance were compensated by >80%. Recordings were performed in ACSF containing (in μM): 1 TTx (Tetrodotoxin; Tocris, 1069), 50 CNQx (6-Cyano-7-nitroquinoxaline-2,3-dione; Tocris, 1045), 25 DL-AP5 (DL-2-Amino-5-phosphonopentanoic acid; Tocris, 0105) and 100 Picrotoxin (Sigma, P1675). Neurons were held at -70 mV, and voltage-ramp protocols from -80 to 80 mV (0.16 mV/ms) from a holding potential of -70 mV were delivered somatically at 0.2 Hz. Voltage clamp recording was performed using a Multiclamp 700A (Molecular Devices) or HEKA EPC10 (HEKA GmBH), data were filtered at 10 kHz and sampled at 20 kHz using pClamp 10.3 or HEKA Pulse. For local perfusion, a theta glass pipette (1 to 1.5 MΩ; Warner instruments, TG200-4) connected to a pressure perfusion system (homemade) was placed above the distal or medial dendrite or in the soma of the recorded neuron, a suction glass pipette was placed downstream from the perfusion pipette to limit the spread of the solution. The extent of the solution spreading was confirmed by perfusing an isotonic sucrose solution (300 mOsm, ion-free) and measuring the offset of the current at the beginning and at the end of the experiments (Womack and Khodakhah, 2002).

### Data Analysis

Electrophysiological data were analyzed using Clampfit 10.3 (Molecular devices) or Igor Pro 6.37 (Wavemetrics). Data are reported as mean ± SEM unless stated otherwise. Statistical significance between groups’ means were tested using a *t*-test or one-way ANOVA. Statistical significance was determined using a *p* < 0.05.

### Reagents

Unless otherwise stated, all salts and reagents were acquired from Sigma–Aldrich.

## Results

### TRPM4 Antibody Validation

For the studies described here, we primarily used two different anti-TRPM4 antibodies that were raised in different species and against immunogens from different regions of TRPM4. Specifically, we used a commercially available rabbit polyclonal antibody (Alomone ACC-044) raised against N-terminal amino acids 5–17 of human TRPM4 (hereafter referred to as rbTRPM4), and the mouse monoclonal L88/86 antibody, which was generated for this project. The L88/86 mAb (hereafter referred to as msTRPM4) was raised against C-terminal amino acids 1040–1214 of human TRPM4. We validated these antibodies by numerous approaches, including immunoblots against HEK293 cells plus/minus expression of human TRPM4 (Supplementary Figures 1A,B), and by immunofluorescence labeling of COS-7 cells expressing FLAG-hTRPM4 (Supplementary Figure 1C) and B16-F10 murine melanoma cells transfected with a shRNA against TRPM4 that expresses GFP as an expression indicator. We found that in B16-F10 2 days after transfection, TRPM4 immunoreactivity is decreased by ≈50% in the GFP positive cells, as compared with the surrounding non-transfected cells (Supplementary Figures 1D–F, arrows). We also employed msTRPM4 and rbTRPM4 antibodies in multiplex immunofluorescence labeling of mPFC in brain sections from postnatal day 35 (P35) mice. We found that these two distinct primary antibodies exhibited virtually identical immunoreactivity patterns (Supplementary Figures 2A–C). A higher magnification view revealed that the distribution of TRPM4 immunolabeling is similar for both antibodies, with a signal overlap over 90% (Supplementary Figures 2D–F). Moreover, we performed tissue immunofluorescence using other anti-TRPM4 commercial antibodies and found a similar somatic and proximal dendritic pattern of immunolabeling (Supplementary Figure 3).

### TRPM4 Expression in Neurons in the mPFC

**FIGURE 1:**
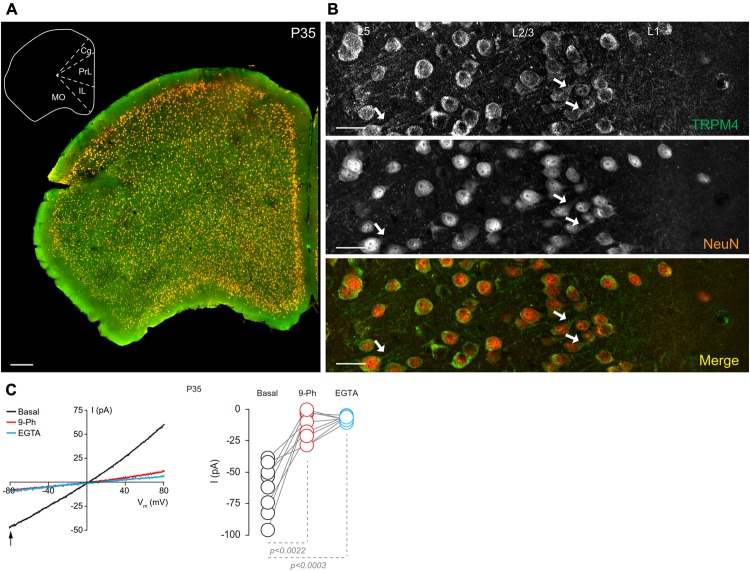
Neuronal expression of TRPM4 in mouse prefrontal cortex (PFC). **(A)** Panoramic view of the frontal cortex showing double immunofluorescence labeling for TRPM4 (green) and NeuN (red) in a coronal mouse brain section at P35. The inset shows a coronal map of this area. **(B)** Confocal images of layers 1 and 2/3 of mPFC showing the expression of TRPM4 (top), NeuN (middle), and the merge of both signals (bottom) showing the expression in the soma and neurites (arrows). Scale bar: **A** = 300 μm; **B** = 40 μm. **(C)** Representative I/V ramps (–80 to 80 mV, 0.16 mV/ms) recorded in the soma of pyramidal neurons from layer 2/3 at mPFC. Black traces represent the basal current, red traces represent the effect of 10 μM 9-Ph (whole bath perfusion), and blue traces represent intracellular EGTA (10 mM). The right panel shows the quantification of the current at –80 mV (arrow) (*n* = 8). All experiments were performed in the presence of glutamatergic (CNQX, AP5) and GABAergic blockers (Picrotoxin).

We assessed the cellular and subcellular expression patterns of TRPM4 immunoreactivity in the mPFC in brain sections prepared from sexually mature male mice at P35 by performing double immunofluorescence labeling for TRPM4 with msTRPM4, and for the post-mitotic neuronal marker NeuN (**Figure 1**). At low magnification, we found that TRPM4 labeling distributed across neurons in the different areas and layers of the frontal cortex, and that most of the NeuN immunoreactive cell bodies are also labeled for TRPM4 (**Figure 1A**). Higher magnification imaging of the mPFC revealed dense TRPM4 immunolabeling in the soma and neurites of neurons layers 2/3 and 5 (**Figure 1B**, arrows); in addition, neurons in layer 1 shows sparse and diffuse TRPM4 and NeuN immunoreactivity in their cell bodies (**Figure 1**). A similar immunolabeling pattern for TRPM4 was obtained with the independent polyclonal antibody rbTRPM4 (Supplementary Figure 4).

To determine whether the expression of TRPM4 as determined by immunolabeling was reflected in functional TRPM4 ionic current, we performed electrophysiological analyses on mPFC neurons in slices prepared from P35 mouse brain. We used I/V ramp protocols in perforated-patch configuration and tested the effect of 10 μM 9-Phenanthrol (9-Ph), which has been shown to be an inhibitor of TRPM4-mediated current (Grand et al., 2008; Burt et al., 2013; Guinamard et al., 2014). We found a basal current of -62.6 ± 7.1 pA (**Figure 1C**, black), with a reversal potential of ∼3 mV, which is inhibited by the 9-Ph (-10.3 ± 3.9 pA; *p* < 0.0022, one-way ANOVA; **Figure 1C**, red). After washout, break-in into the whole cell configuration allows the diffusion of the high EGTA (10 mM) intracellular solution into the neuron, at which point we observed a complete reduction in the current (-7.22 ± 0.6 pA, *p* < 0.0003, one-way ANOVA), thus confirming the CAN nature of this current (**Figure 1C**, blue).

### TRPM4 Expression in Different Cell Types

**FIGURE 2:**
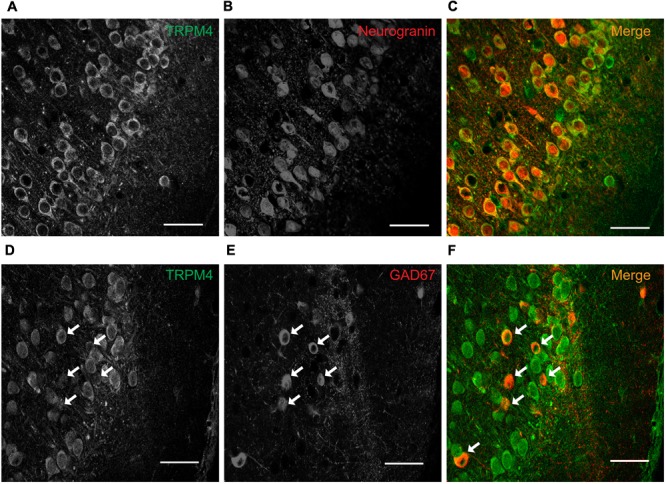
TRPM4 is expressed in pyramidal neurons and interneurons in layers 2/3 of the mPFC. Confocal images from double immunofluorescence labeling for **(A)** TRPM4 (green), **(B)** Neurogranin (red), and **(C)** the merged signals in a coronal mouse brain section at P35. Confocal images from double immunofluorescence labeling for **(D)** TRPM4 (green), **(E)** GAD67 (red), and **(F)** the merge of both signals in a coronal mouse brain section at P35. Arrows point to expression on interneurons. Scale bar = 40 μm.

To characterize the neuronal cell types exhibiting immunolabeling for TRPM4, we performed double label immunofluorescence using combination of msTRPM4 and anti-Neurogranin (to immunolabel excitatory neurons), and msTRPM4 and anti-GAD67 (to label inhibitory neurons) antibodies in brain sections from P35 mice containing mPFC. Our results showed that, as expected, most of the neurons in mPFC layers 2/3 are Neurogranin-positive (**Figures 2A–C**), and that these cells are evenly distributed across the layers. As expected, the GAD67-expressing cells are more sparsely distributed across the layers (**Figures 2D–F**, arrows). Both populations of neurons had robust immunolabeling for TRPM4 (**Figures 2A–C**), suggesting that TRPM4 is expressed in both excitatory and inhibitory neurons.

### TRPM4 Distribution in mPFC Neurons in Layers 2/3

**FIGURE 3:**
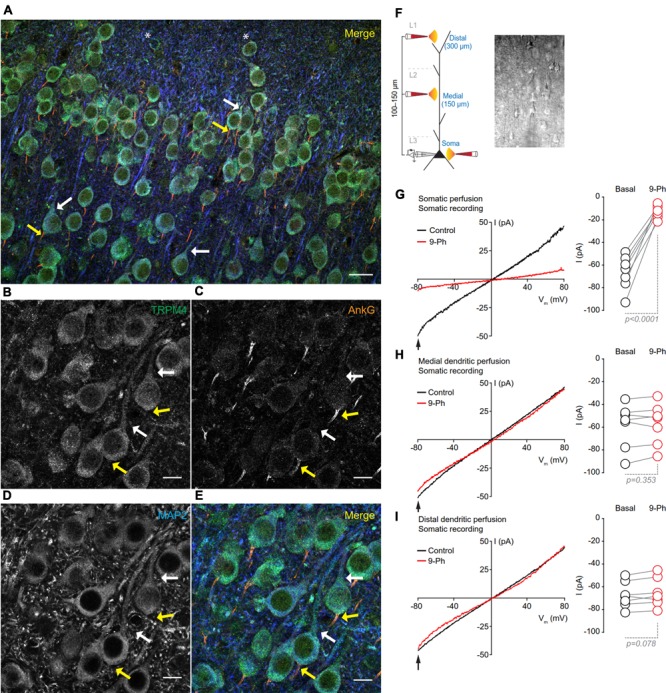
TRPM4 expression is restricted to the somatodendritic region of the pyramidal neurons at mPFC layers 2/3. **(A)** Confocal images from triple immunofluorescence labeling for TRPM4 (green), MAP2 (blue), and AnkG (orange) in the mPFC in a coronal mouse brain section at P35. Magnification of layers 2/3 showing **(B)** TRPM4, **(C)** MAP2, **(D)** AnkG and **(E)** merge of the three signals, white arrows indicate TRPM4 and MAP2 co-expression in proximal dendrites, yellow arrows indicate AnkG expression in the AIS, which does not contain detectable TRPM4 immunolabeling. Scale bar: **A** = 20 μm; **B–E** = 10 μm. **(F)** Schematic figure showing the areas of drug perfusion and a DIC picture of the recorded area. Representative I/V curves (–80 to 80 mV, 0.16 mV/ms) recorded in the soma using local somatic **(G)**, medial, **(H)**, and distal dendrite **(I)** drug perfusion; black traces represent the basal current, red traces represent the effect of 10 μM 9-Ph. Right panels showed the quantification of the currents at –80 mV (arrow) (*n* = 7). All experiments were performed in the presence of glutamatergic (CNQX, AP5) and GABAergic blockers (Picrotoxin).

To determine the subcellular localization of TRPM4 in neurons in layers 2/3 of the mPFC, we performed triple label immunofluorescence against TRPM4 (msTRPM4, green), AnkG (axon initial segment, AIS, orange) and MAP2 (soma and dendrites, blue). We found overlap of the TRPM4 and MAP2 immunolabels on the soma and proximal dendrites across most neurons (**Figures 3A,B,D,E**, white arrows), but little or no overlap of TRPM4 with AnkG (**Figures 3A,B,C,E**, yellow arrows). We also observed a few longer processes expressing both TRPM4 and MAP2, but we were unable to trace their origin to any cell body. We also observed a few neurons in layer 1 that exhibited labeling for both TRPM4 and MAP2 (asterisks).

To test whether the specific pattern of expression of TRPM4 is correlated with TRPM4-like currents, we performed somatic I/V ramp protocols (-80 to 80 mV) in perforated-patch configuration and locally applied 10 μM 9-Ph in three different areas; soma, medial and distal dendrite (layer 1) of pyramidal neurons (**Figure 3F**). We found that somatic perfusion of 10 μM 9-Ph reduces the basal current (0 min = -66.5 ± 5.7 pA, 5 min = -12.9 ± 2.1 pA, *p* < 0.0001, *t*-test; **Figure 3G**) with a reversal potential of ∼2 mV. However, the application of 9-Ph to the region containing medial (0 min = -58.7 ± 7.4 pA, 5 min = -57.2 ± 6.9 pA, *p* = 0.353, *t*-test; **Figure 3H**) or distal (0 min = -67.3 ± 4.4 pA, 5 min = -65.2 ± 4.8 pA, *p* = 0.078, *t*-test; **Figure 3I**) dendrites did not affect the TRPM4-like current, suggesting that, similar to the TRPM4 immunolabeling, functional TRPM4 is located, in the soma, and is not detectable in the medial and distal dendrites.

### TRPM4 Expression during Postnatal Development

**FIGURE 4:**
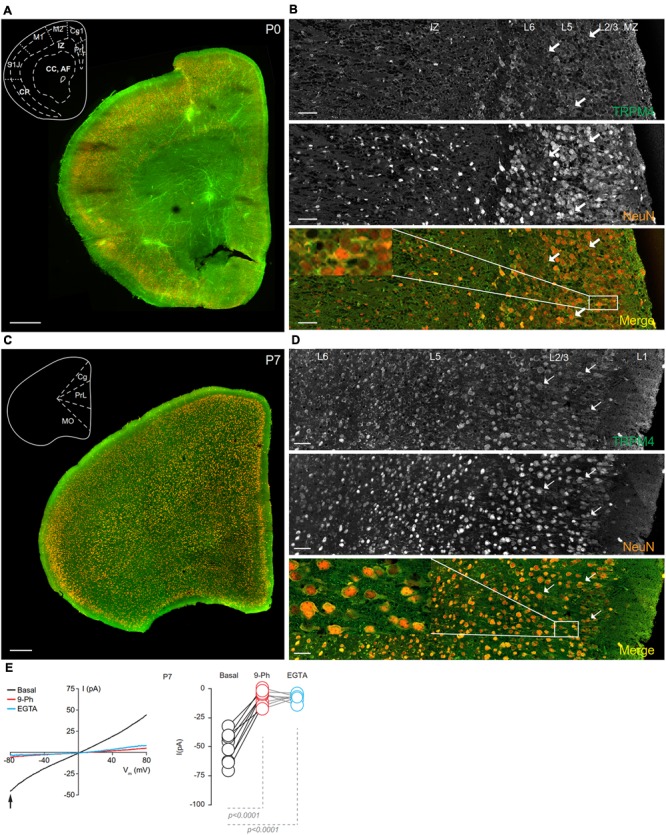
TRPM4 expression at P0 and P7 in the mPFC. **(A)** Panoramic view of the frontal cortex in a coronal mouse brain section at P0, showing double immunofluorescence labeling or TRPM4 (green) and NeuN (orange). The inset shows a coronal map of the area. **(B)** Confocal images from the mPFC at P0 showing TRPM4 (top panel), NeuN (middle), and the merged signals (bottom). **(C)** Panoramic view of the frontal cortex in a coronal mouse brain section at P7, showing double immunofluorescence labeling for TRPM4 (green) and NeuN (orange), inset shows a coronal map of the area. **(D)** Confocal images from cortical layers of mPFC at P7 showing TRPM4 (top), NeuN (middle) and merged signals (bottom). Arrow shows expression in soma and neurites. Scale bar: **A,C** = 300 μm; **B,D** = 40 μm. **(E)** Representative I/V curve (–80 to 80 mV, 0.16 mV/ms) in perforated-patch configuration indicating the effect of 10 μM 9-Ph (whole bath perfusion). Black traces represent the basal current, red traces represent the effect of 10 μM 9-Ph and blue traces represent the effect of 10 mM EGTA. Right panel showed the quantification of the current at –80 mV (arrow) in P7 mice (*n* = 8). All experiments were performed in the presence of glutamatergic (CNQX, AP5) and GABAergic blockers (Picrotoxin).

To determine the cellular expression and subcellular localization of TRPM4 during postnatal development, we performed double immunofluorescence for TRPM4 and NeuN in brain sections from frontal cortex from mice at postnatal ages P0, P7, P14, and P90. We found that immunolabeling for msTRPM4 was already present at P0, with labeling distributed throughout the cortical layers. Most of the overlapping msTRPM4 and NeuN labeling was present in primary somatosensory, primary motor, and cingulate cortex. In these areas, robust TRPM4 immunolabeling occurs in layers 2/3, with labeling decreasing in intensity through layers 5 and 6. In the prelimbic and infralimbic area, TRPM4 immunolabeling is weak and most of it occurs in layers 2/3 (**Figure 4A**). A higher magnification view of prelimbic and infralimbic area showed densely packed neurons with somatodendritic TRPM4 immunolabeling (**Figure 4B**, arrows an inset) with a few and sparse labeling in the intermediate zone (IZ). We were not technically able to record currents in neurons from slices prepared from P0 mouse brain.

TRPM4 immunoreactivity at P7 is higher than at P0, and also exhibited a broader distribution throughout the cortical regions, with no clear differences in the expression between layers in most of the areas. The primary motor cortex, frontal area 3, and insular regions exhibited increased overlap of TRPM4 and NeuN labeling, particularly in layers 2/3 (**Figure 4C**). A magnification of the mPFC (prelimbic area) showed TRPM4 immunoreactivity in the cell body and in the proximal neurites (short processes) (**Figure 4D**, arrows and inset); TRPM4 labeling is qualitatively similar in all layers, but with few cells labeled in layer 1. I/V ramp protocols performed using perforated-patch configuration from mPFC neurons in P7 brain slices showed a basal current of -50.8 ± 4.8 pA (**Figure 4E**, black), with a reversal potential (V_rev_) of ∼5 mV, which is inhibited by the TRPM4 inhibitor 9-Ph (-7.2 ± 2.3 pA; *p* < 0.0001, one-way ANOVA; **Figure 4E**, red). After washout, break-in into the whole cell configuration allows for the diffusion of the high EGTA (10 mM) intracellular solution into the neuron, at which point we observed a complete reduction in the current (-7.3 ± 1.2 pA; *p* < 0.0001, one-way ANOVA), thus confirming the CAN nature of this current (**Figure 4E**, blue).

**FIGURE 5:**
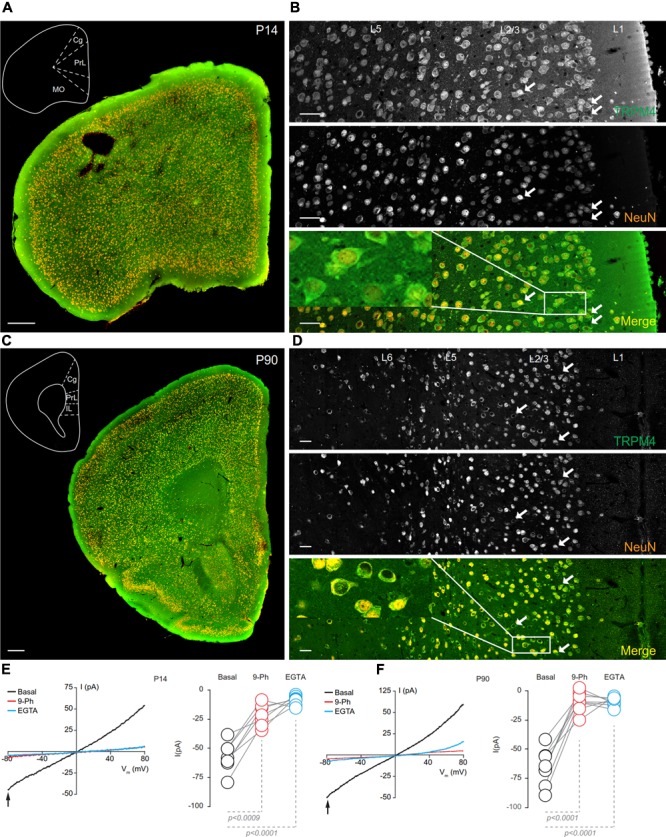
TRPM4 expression at P14 and P90 in the mPFC. **(A)** Panoramic view of the frontal cortex in a coronal mouse brain section at P14, showing double-immunofluorescence labeling for TRPM4 (green) and NeuN (orange). The inset shows a coronal map of the area. **(B)** Confocal images from the mPFC at P14 showing TRPM4 (top panel), NeuN (middle), and the merged signals (bottom). **(C)** Panoramic view of the frontal cortex in a coronal mouse brain section at P90, showing double immunofluorescence labeling for TRPM4 (green) and NeuN (orange), inset shows a coronal map of the area. **(D)** Confocal images from cortical layers of mPFC at P90 showing TRPM4 (top), NeuN (middle), and the merged signals (bottom). **(E)** Representative I/V curves (–80 to 80 mV, 0.16 mV/ms) in perforated-patch configuration showing the effect of 10 μM 9-Ph (whole bath perfusion), right panel showed the quantification of the current at –80 mV (arrow) in P14 (*n* = 7). **(F)** Representative I/V curve (–80 to 80 mV, 0.16 mV/ms) in perforated-patch configuration indicating the effect of 10 μM 9-Ph (whole bath perfusion), right panel showed the quantification of the current at P90 (*n* = 8). The arrows in all images shows expression in soma and neurites, black arrows in the plots show the voltage at which currents were measured (–80 mV). Black traces represent the basal current, red traces represent the effect of 10 μM 9-Ph and blue traces represent the effect of 10 mM EGTA. All experiments were performed in the presence of glutamatergic (CNQX, AP5) and GABAergic blockers (Picrotoxin). Scale bar: **A,C** = 300 μm; **B,D** = 40 μm.

At P14, overlapping TRPM4 and NeuN labeling was found throughout the cortical regions (**Figure 5A**). At this stage, we observed an increase in immunolabeling of neuropil, and layer 1 exhibited a sparse distribution of neurons labeling for both TRPM4 and NeuN, contrary to layers 2/3 where it was more extensive. TRPM4 immunolabeling was present with a relatively even distribution in all areas, with enrichment in primary motor and agranular insular cortex. A higher magnification view of the prelimbic area confirmed the distribution of TRPM4 immunolabeling in all layers, in which labeling is restricted to soma and to the proximal dendrite (**Figure 5B**, arrows and inset). Ramp protocols performed using perforated patch-clamp on mPFC neurons in P14 mouse brain slices exhibited a basal current of -57.1 ± 4.9 pA (**Figure 5E**, black), similar to P7, the reversal potential was ∼5 mV with no rectification, the application of 9-Ph reduces the current (-21.3 ± 3.6 pA; *p* < 0.0009, one-way ANOVA, **Figure 5E**, red), and whole-cell recordings in high EGTA showed a reduction in the current (-8.7 ± 1.5 pA; *p* < 0.0001, one-way ANOVA), again confirming its CAN nature (**Figure 5E**, blue).

In brain sections prepared from P90 mice, we observed TRPM4 immunolabeling, which is evenly distributed through the cortical areas; most of TRPM4 immunoreactive cells coexpressed NeuN (**Figure 5C**). A higher magnification of the prelimbic area exhibited an enrichment for TRPM4 immunolabeling in layers 2/3, and 5, with the TRPM4 immunolabeling localized to the somatodendritic region (**Figure 5D**, arrows and inset); similar to earlier development stages, we found sparse labeling in layer 1. The ramp protocols performed in perforated patch-clamp on mPFC neurons in P90 mouse brain slices showed a basal current of -65.4 ± 5.4 pA (**Figure 5F**, black) with a reversal potential of ∼3 mV with no rectification, the application of 9-Ph reduces the current (-8.8 ± 2.9 pA; *p* < 0.0001, one-way ANOVA, **Figure 5F**, red). Moreover, the high EGTA intracellular solution completely abolished the current (-9.1 ± 1.3 pA, *p* < 0.0001, one-way ANOVA) after break-in the whole-cell configuration (**Figure 5F**, blue). Together, these results indicate that TRPM4 expression and TRPM4-like currents are present in pyramidal neurons of the mPFC from early postnatal developmental stages and this current is active in resting non-stimulated condition.

## Discussion

In this report, we characterized the cellular and subcellular pattern expression of the TRPM4 ion channel in the mPFC during postnatal mouse development, using multiple label immunofluorescence, and employing highly validated antibodies. We demonstrated that TRPM4 immunolabeling increases during postnatal development up to P35, and that at all stages immunolabeling is restricted to the soma and proximal dendrites of the pyramidal neurons. We also found that both interneurons and pyramidal neurons have immunolabeling for TRPM4. These patterns of expression suggest a role for TRPM4 channels in the physiology of these neurons, and particularly, given the functional properties of TRPM4, in their intrinsic excitability.

A previous study using immunofluorescence labeling of adult mouse brain sections with an anti-TRPM4 antibody distinct from those used here showed a somatic pattern of TRPM4 immunolabeling in pyramidal neurons in layer 5 of mPFC (Lei et al., 2014); however, no other layers were studied. In this study, we found widespread somatodendritic labeling for TRPM4 throughout all cortical layers, with an enrichment in layers 2/3 at early postnatal ages, and becoming more uniform across the layers after age P35. The TRPM4 immunolabeling we observed in the mPFC is generally consistent with the cellular pattern of TRPM4 mRNA expression in the Allen Brain Atlas (Allen Institute for Brain Science, 2017b), which shows a moderate and widespread neuronal expression in the frontal cortical area. The subcellular pattern of TRPM4 immunolabeling in mPFC neurons presented here is similar to that shown previously (Kim et al., 2013) in cerebellar lobe IX Purkinje neurons, where TRPM4 immunolabeling was also confined to the somatodendritic region, with no apparent signals in the axon. Another study demonstrated somatic localization of TRPM4 immunoreactivity in neurons of the accessory olfactory bulb, where TRPM4 was suggested to participate in the prolonged firing seen after EPSC-like stimulation (Shpak et al., 2012). Interestingly, while the somatodendritic localization of TRPM4 shown here is also seen in spinal cord neurons, there is additional axonal immunolabeling for TRPM4 that is low under control conditions, but that increases after inflammatory spinal cord damage (Schattling et al., 2012).

Our findings support that TRPM4 is primarily expressed in the soma and proximal dendrites of the pyramidal neurons in the mPFC layer 2/3. These results are supported by the electrophysiological data showing that somatic perfusion of 9-Ph reduces the non-selective cation current, while perfusion at sites corresponding to the medial or distal dendrites does not. The basis for the differences between what we observed here in mPFC, and the axonal TRPM4 immunolabeling patterns previously reported in the spinal cord is not known. While this could be a biological difference between mPFC pyramidal neurons and spinal cord neurons, differences in the accessibility of the antibody into the axon and more distal dendrites in the different preparations could also be a contributing factor. That we performed our immunolabeling in mPFC under a variety of conditions, employing antigen retrieval, a different fixative solution (acetone) and different antibodies, we did not find expression in the axon initial segment or in the axon proper, suggests that the levels of TRPM4 in these regions is absent, or sufficiently low to be undetectable. Taken together with the prominent colocalization of the immunoreactivity for TRPM4 with the somatodendritic marker MAP2 leads us to conclude that TRPM4 expression is primarily restricted to the soma and proximal dendritic region of mPFC pyramidal neurons. In contrast, the interneurons in the mPFC express TRPM4 primarily in the soma, with little if any immunolabeling in the dendrites. Investigating the mPFC interneuron types that express TRPM4 will allow us to better understand the contribution of TRPM4 to local circuit function, as different interneurons target different regions of mPFC pyramidal neurons (i.e., axon, soma, or dendrites) modulating their excitability, firing probability, network activity and synaptic integration (Markram et al., 2004). Interestingly, in all the postnatal stages studied, we found a few cells labeled for TRPM4, but not co-labeled for any neuronal marker. These results suggest that brain cell types other than neurons, such as glial cells, may also express TRPM4. In this context, Cáceres et al. (2015) demonstrated that astrocytes in primary culture express TRPM4 at focal adhesions. As such, the role of TRPM4 channels in these non-neuronal cell types, and in neuron-glia interaction, constitute an interesting question to examine.

To date, only transcriptomic data is available on the developmental expression of TRPM4, and this is only for human brain. This reveals TRPM4 mRNA expression is relatively constant throughout dorsolateral PFC, an area homologous to the mPFC in mice (Bicks et al., 2015; Allen Institute for Brain Science, 2017a). To our knowledge, this study is the first description of TRPM4 protein expression in mouse PFC during postnatal development. Our results show that in mice, TRPM4 immunoreactivity increases in the frontal cortex as postnatal development progresses up to P35. Moreover, we found that the subcellular localization of TRPM4 immunolabeling in pyramidal neurons changes from a primarily somatic pattern, with labeling of few proximal dendrites, to a somatodendritic pattern with more extensive labeling of proximal dendrites at later developmental stages. These data are supported by electrophysiological data showing a 9-Ph sensitive CAN current compatible with TRPM4 also increases during this period. Interestingly, the TRPM4-like current is active at resting membrane potentials, suggesting that this current may be participating in the modulation of the resting membrane potential in a Ca^2+^ -dependent manner. In this context, the range of sensitivity for calcium goes from 0.05 to 10 μM, moreover, at resting condition, Ca^2+^_i_ levels vary between 0.05 and 0.3 μM in pyramidal neurons (Wojda et al., 2008), therefore the Ca^2+^ concentration is enough to activate at least a fraction of TRPM4 at resting membrane potentials.

We determined the specificity of the novel monoclonal antibody developed for these studies by several methods including immunoblots, immunofluorescence labeling of a cell line that endogenously expresses TRPM4 plus/minus shRNA-mediated TRPM4 knockdown, and double immunofluorescence labeling of brain sections using two different antibodies raised in different species, and against different regions of TRPM4. In our knockdown experiments, we observed a substantial reduction in the immunofluorescence labeling signal obtained with the msTRPM4 monoclonal antibody in the shRNA expressing cells. The sample preparation conditions for immunofluorescence labeling of this cell line, and of the mouse brain sections, are similar and provides support for the specificity of immunofluorescence labeling using this antibody. We also showed a strong overlap (≈90%) between immunolabeling signals obtained in mouse brain sections using two different anti-TRPM4 antibodies, raised in different species and against two different regions of TRPM4. This supports that both antibodies bind to the same target, and serves as an additional demonstration of the specificity of the antibody (Rhodes and Trimmer, 2006; Lorincz and Nusser, 2008).

The expression levels and subcellular localization of ion channels is critical for defining the excitable properties of neurons, in determining local membrane resistance and membrane potential. During electrical activity, dynamic changes in the distribution of ion channels contribute to plasticity of synaptic integration and intrinsic excitability. As one example, elevated neuronal activity leads to changes in the subcellular localization and voltage-dependent activation of neuronal Kv2.1 channels, which then impacts excitability (Cerda and Trimmer, 2010, 2011). Similarly, action potential generation and backpropagation, which requires a high density of sodium channels in the axon initial segment (Kole et al., 2008) is dynamically regulated by activity-dependent changes in sodium channel expression and localization in the axon initial segment (Grubb and Burrone, 2010; Kuba et al., 2010). Furthermore, Kv1 channels located in the AIS allow pyramidal neurons in layer 5 to integrate slow threshold signals controlling the action potential and synaptic coupling (Kole et al., 2007), and these are also subject to activity-dependent plasticity (Kuba et al., 2015). Pyramidal neurons express HCN1 channels in an increasing gradient throughout the dendrites, this determine their properties of synaptic integration and excitability (Lörincz et al., 2002; Arimitsu et al., 2009). The subcellular localization of TRPM4 in mFPC neurons may contribute to compartment-specific effects on intrinsic excitability or synaptic integration by controlling local depolarization during synaptic activity. The Ca^2+^ -dependency of TRPM4 activity could provide an additional mechanism for the control of the excitability.

## Author Contributions

DR, IS, JH-T, OC, and EL-S conducted the experiments; AP and JT contributed the new reagents; DR, EL-S designed the research and wrote the manuscript; DR, OC, JT, and EL-S edited the manuscript.

## Conflict of Interest Statement

The authors declare that the research was conducted in the absence of any commercial or financial relationships that could be construed as a potential conflict of interest.
