# Supplementary figures and images for: Subcellular Localization and Activity of TRPM4 in Medial Prefrontal Cortex Layer 2/3
Source: Front Cell Neurosci. 2018 Jan 30;12:12. doi: 10.3389/fncel.2018.00012 (PMC5797675; doi:10.3389/fncel.2018.00012)

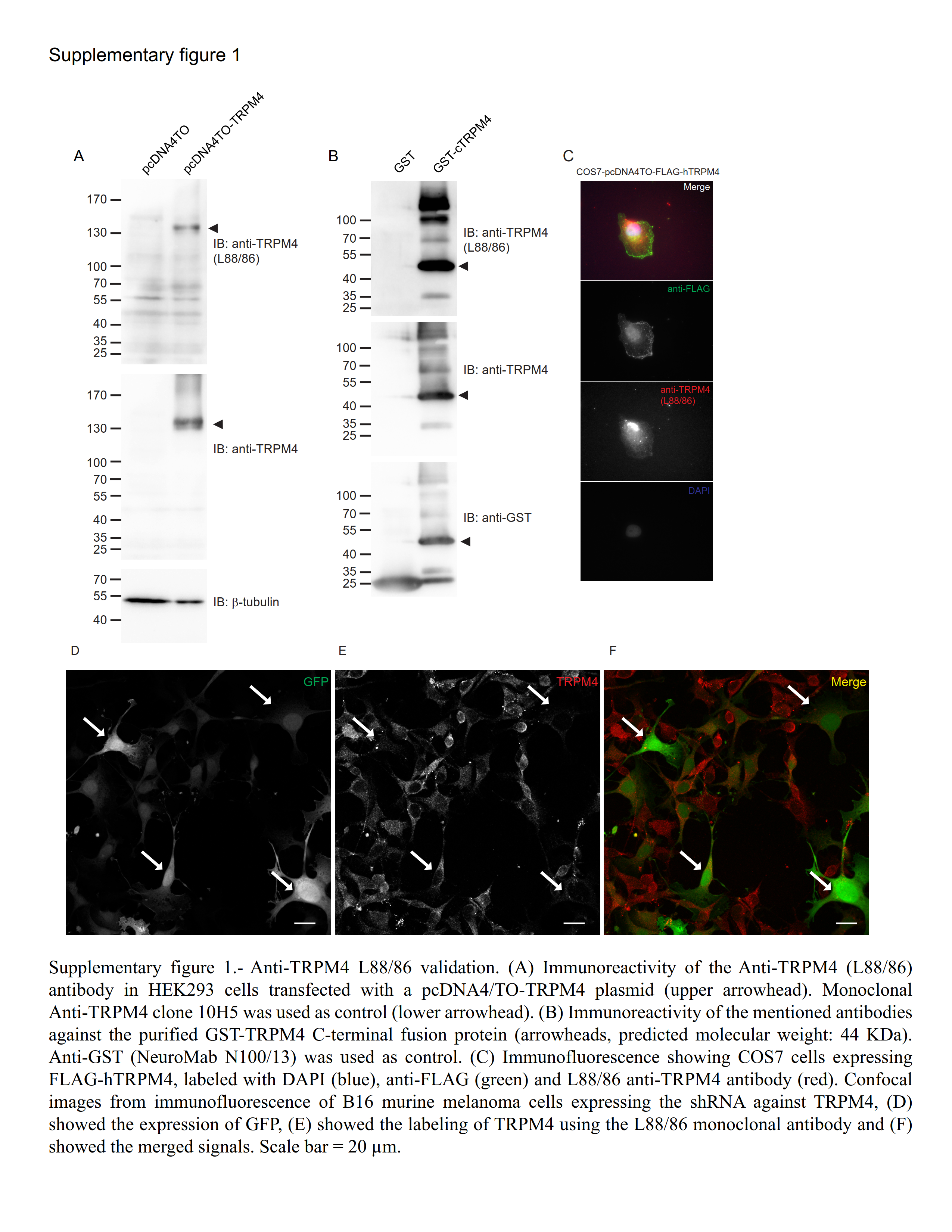

Supplement: Supplementary file 1 [file Image_1.tif]

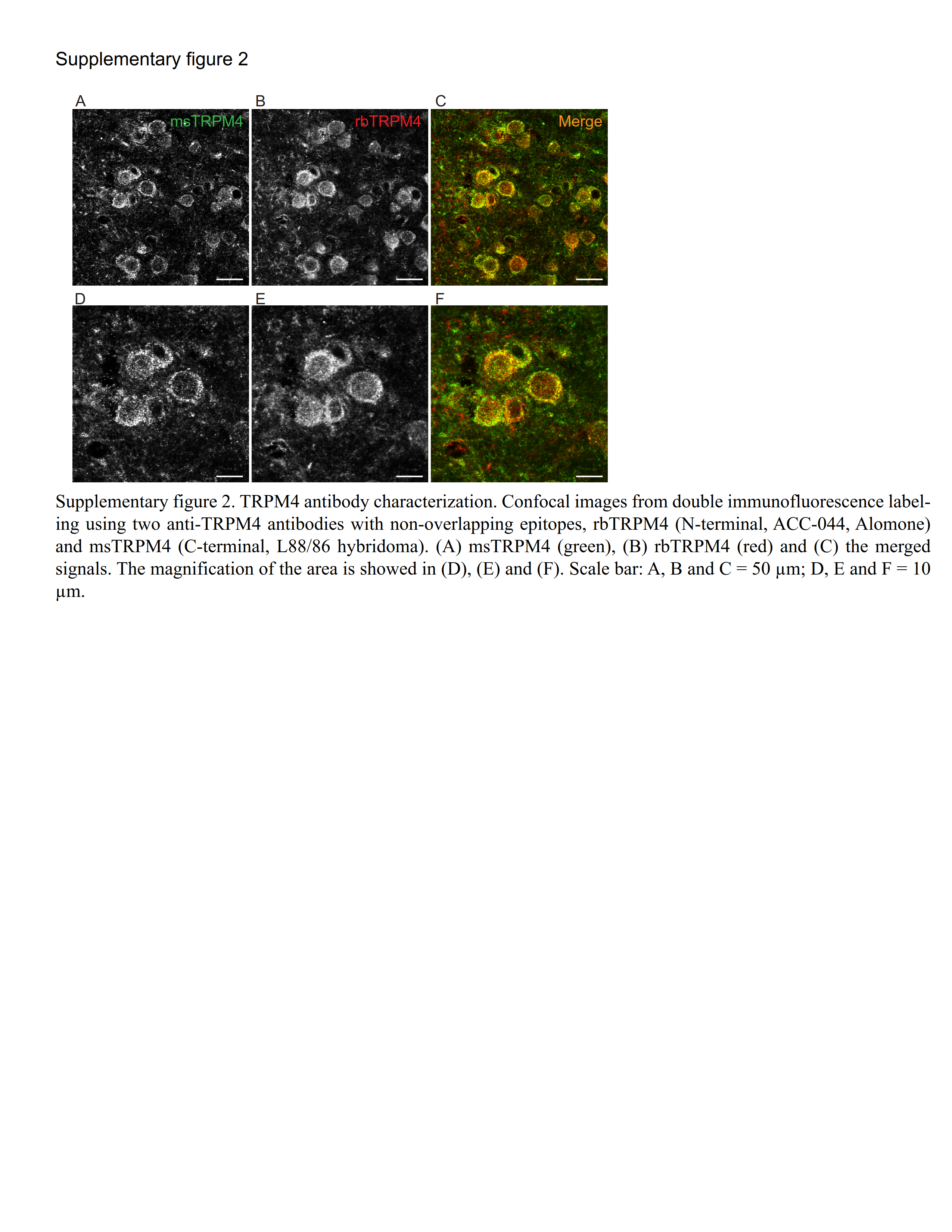

Supplement: Supplementary file 2 [file Image_2.tif]

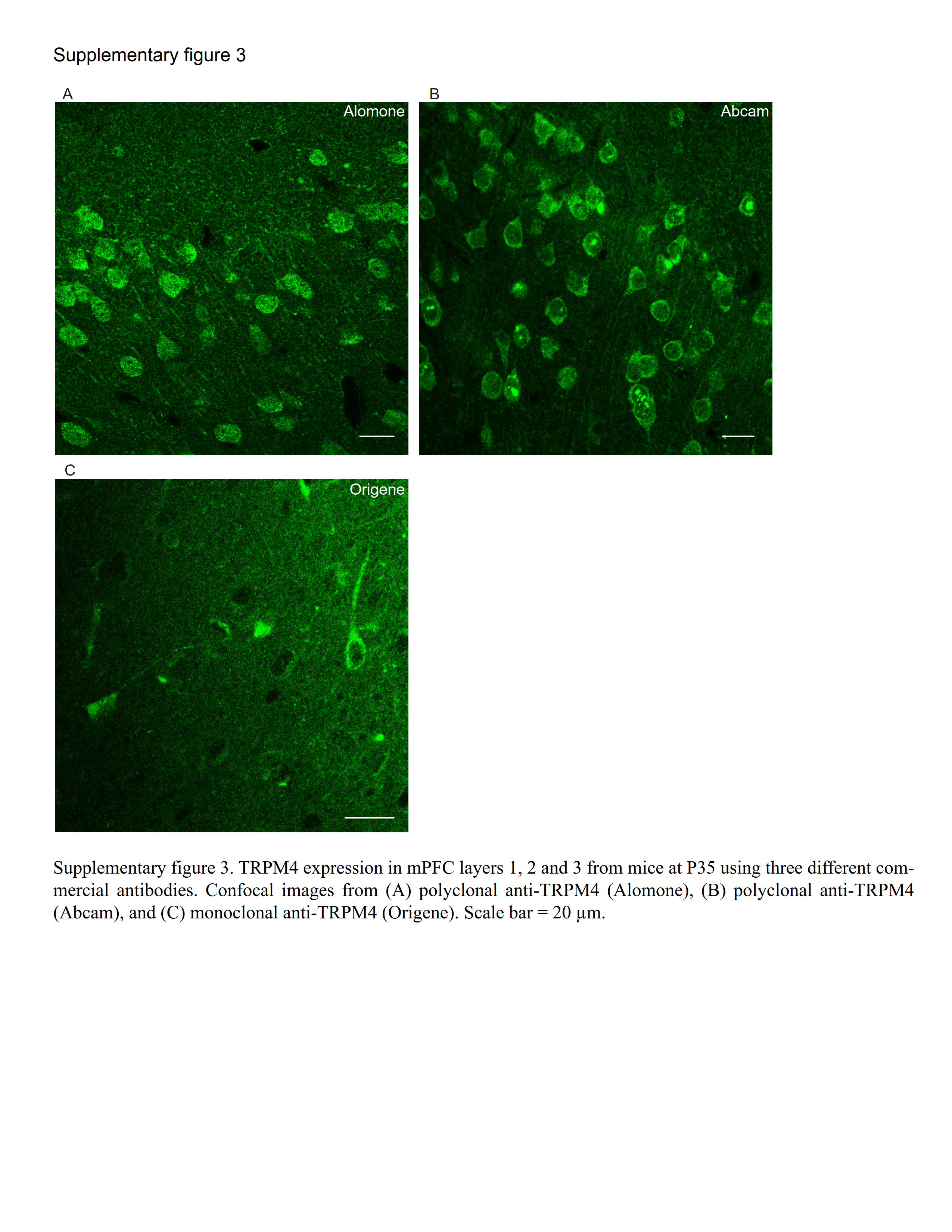

Supplement: Supplementary file 3 [file Image_3.tif]

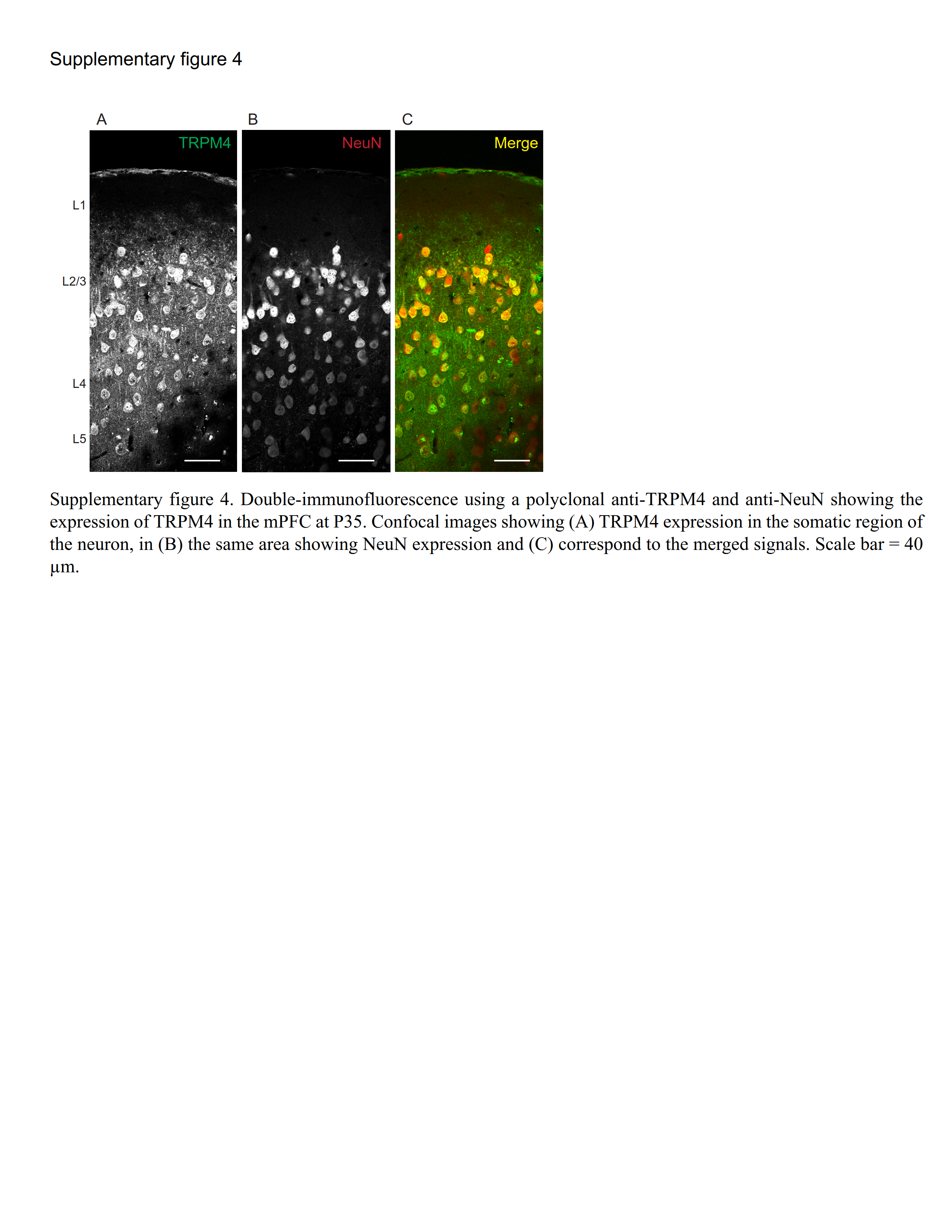

Supplement: Supplementary file 4 [file Image_4.tif]
